# Bioremediation Options for Heavy Metal Pollution

**DOI:** 10.5696/2156-9614-9.24.191203

**Published:** 2019-11-27

**Authors:** Meena Kapahi, Sarita Sachdeva

**Affiliations:** 1 Department of Biotechnology, Manav Rachna International Institute of Research and Studies, Faridabad, India; 2 Department of Chemistry, Manav Rachna University, Faridabad, India

**Keywords:** bioremediation, microorganisms, heavy metals, contaminants, environment, organic matter, biosorption

## Abstract

**Background.:**

Rapid industrialization and anthropogenic activities such as the unmanaged use of agro-chemicals, fossil fuel burning and dumping of sewage sludge have caused soils and waterways to be severely contaminated with heavy metals. Heavy metals are non-biodegradable and persist in the environment. Hence, remediation is required to avoid heavy metal leaching or mobilization into environmental segments and to facilitate their extraction.

**Objectives.:**

The present work briefly outlines the environmental occurrence of heavy metals and strategies for using microorganisms for bioremediation processes as reported in the scientific literature.

**Methods.:**

Databases were searched from different libraries, including Google Scholar, Medline and Scopus. Observations across studies were then compared with the standards for discharge of environmental pollutants.

**Discussion.:**

Bioremediation employs microorganisms for removing heavy metals. Microorganisms have adopted different mechanisms for bioremediation. These mechanisms are unique in their specific requirements, advantages, and disadvantages, the success of which depends chiefly upon the kind of organisms and the contaminants involved in the process.

**Conclusions.:**

Heavy metal pollution creates environmental stress for human beings, plants, animals and other organisms. A complete understanding of the process and various alternatives for remediation at different steps is needed to ensure effective and economic processes.

**Competing interests.:**

The authors declare no competing financial interests.

## Introduction

**Figure 1:**
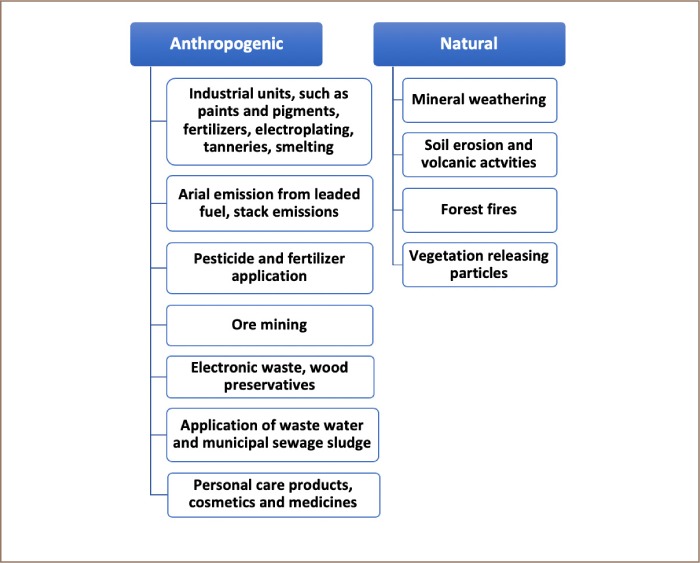
Sources of heavy metals

Heavy metal pollution is a serious concern due to hazardous impacts at even very small concentrations. Heavy metals are non-biodegradable, bioaccumulate in tissues and are biomagnified along with the trophic levels.^1^Weathering of geological bedrock and volcanic eruptions can discharge heavy metals into the surrounding environment.^2^ The type of heavy metals released from the rock substratum depends on its composition and other factors such as the inherent chemistry of the bedrock/soil, climate, nature, and composition of the soil and other anthropogenic activities in the region.^2^,^3^ Subsequent releases and the entry of heavy metals into the food chain depends on their concentration and uptake by the local flora and fauna. Atmospheric deposition has also been reported to be one of the major causes of deposits in urban and sub-urban areas. Heavy metal sources can be categorized as shown in Figure 1.

Heavy metal pollution occurs directly from industries (tannery, electroplating, dyeing, mining), agricultural fields, sewage sludge, and waste treatment plants. Recent studies have established that the long-term use of untreated wastewater from industrial sources can adversely affect water quality, making it unfit for human consumption.^2^ Untreated industrial wastewater is often colored, frothy, and contains hazardous chemicals including heavy metals, toxic dyes, acids, alkalis, and other toxic chemicals.^4^ The resulting pollution leads to hazardous impacts on the health of occupants/residents and occupational health hazards for workers.^5^ The electroplating industry releases hazardous wastewater laden with heavy metals.^6^ Heavy metals such as chromium (Cr) and nickel (Ni) discharged in untreated effluents by electroplating plants have been reported to surpass permissible limits.^6–10^ Heavy metals like copper (Cu), Cr, iron (Fe), manganese (Mn), and zinc (Zn) are present in tannery wastewater.^11^ Various studies have analyzed the quality of industrial wastewater and groundwater supplies in terms of heavy metals compared with standards for discharge of environmental pollutants *(Table 1)*.

**Table 1:** Sources of Heavy Metal Contamination in Water

**Element**	**References**										

	**12**	**13**	**14**	**15**	**16**	**17**	**18**	**19**	**20**	**21**	**22**
**As**					•	x			x		
**Cd**	•	•		x	x		x	x		x	x
**Cr**	•	x		x			x	x		x	
**Cu**	x	x	•	•	•		•	•	x	x	x
**Fe**				x	•				x	x	
**Hg**					•						
**Mn**				•							
**Ni**	•	x	•	x			•	•		x	
**Pb**			•		x		•	•	x	x	x
**Zn**			•	•	•		•	•	x	x	x
**Source**	Textile mill effluent	Electroplating industry wastewater sample	Ganga river water quality	Ground water quality	Ground water quality	Water supply	Irrigation water	Irrigation water	Irrigation water	Lake water	Irrigation water

x indicates the concentration of heavy metals found above permissible limits;

• is the concentration of heavy metals found below permissible limits.

Bureau of Indian Standards for drinking water (BIS-10500-2012): Accepted value in mg/L (permissible limits in the absence of alternate source in mg/L) – As, 0.01 (0.05); Cd, 0.003 (no relaxation); Cu: 0.05 (1.5); Pb,: 0.01 (no relaxation); Hg, 0.001 (no relaxation); Fe: 0.3 (no relaxation); Ni: 0.02 (no relaxation); Cr: 0.05 (no relaxation); Zn: 5 (15); Mn: 0.1 (0.3).

World Health Organization standards for surface waters (μg/L): As, 10; Cr: 50; Cd, 3; Cu: 2000; Ni: 70; Pb,10; Hg, 01.

Abbreviations: As, arsenic; Cd, cadmium; Cr, chromium; Cu, copper; Fe, iron; Hg, mercury; Mn, manganese; Ni, nickel; Pb, lead; Zn, zinc.

Various reports have demonstrated that industrial wastewater contains heavy metals beyond permissible limits for drinking water or surface/irrigation water. Wastewater containing heavy metals (above permissible levels) is used to irrigate fields in various parts of India. Application of heavy metal-contaminated water in agricultural fields has led to their bioaccumulation in crops and associated food chains. Indirect heavy metal pollution results from contaminated surface or groundwater and rainwater. Rivers are one of the most important resources for fresh water and are severely affected by pollution sources.^23^ According to the report ‘Status of trace and toxic metals in Indian rivers’, out of 414 river water quality stations across various rivers in India, 57 stations have been found to contain two or more heavy metals beyond permissible limits.^24^ The situation demands immediate action and remediation of contaminated rivers. The chances of exposure to heavy metals have increased due to increased use in the technology, domestic, industrial, and agricultural sectors.^2^ Heavy metals' effects depend largely upon their chemical nature. Inorganic arsenic (As) compounds are readily absorbed and interfere to a greater extent with cellular reactions compared to the organic forms due to their poor cellular absorption.^25^ Heavy metals have been reported to attach themselves to protein binding sites and remove the original metals, causing toxicity and cellular malfunctioning.^26^

Different exposure routes, including dermal (through the skin), ingestion (food or drink), and inhalation (as dust or fume) have been studied, along with the effects of heavy metals, mechanism of toxic action, impacts, and conventional and bioremediation techniques. A number of studies have examined various bioremediation techniques including algae, bacteria, and fungi as biosorbents. The current study aims to provide insight into the environmental occurrence and sources of heavy metals as published in the scientific literature.

## Methods

The present study was conducted by searching databases from different libraries: Google Scholar, Medline and Scopus. Studies, irrespective of the place/area of research, were restricted to publications available in English and those published in the last twenty years. The search outcomes were collected, studied and thoroughly mined as applicable to the study objectives. Observations across studies were compared with standards for the discharge of heavy metals.

**Figure 2:**
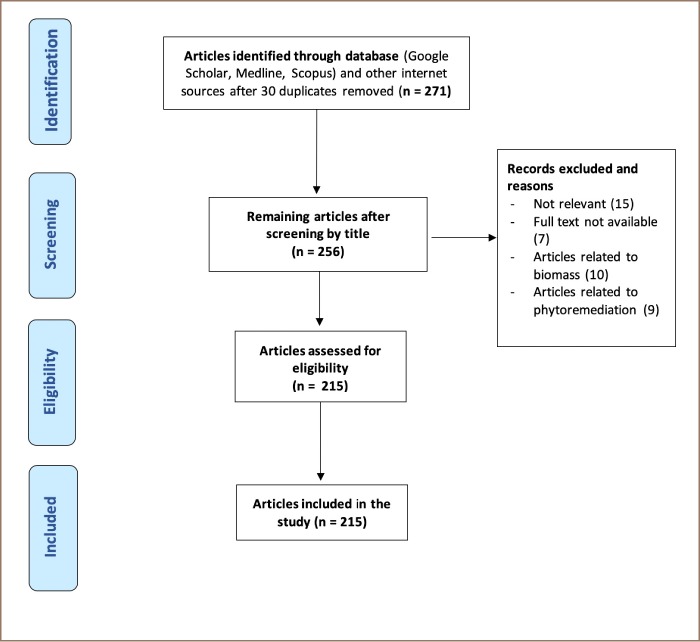
Flow diagram of article selection process

A total of 301 articles or records were searched in various databases. Most of the articles were identified from electronic bibliographic sources. After the initial adjustment with duplicates (n = 29), 271 articles were further screened on the basis of title (n = 15), relevance (n = 15), and availability of full text (n = 07). Articles related to biosorption with biomass and phytoremediation were discarded (n = 19). The remaining articles (n = 215) were assessed for eligibility and included in the study *(Figure 2)*.

## Results

Table 2 demonstrates some of the toxic heavy metals, their occurrence/applications, exposure routes and toxicity mechanisms.

**Table 2:** Environmental Occurrence, Routes of Exposure and Toxicity Profile of Toxic Heavy Metals

**Toxic heavy metals**	**Environmental occurrences**	**Anthropogenic sources/commercial applications**	**Exposure routes**	**Effects**	**Toxicity and mechanisms**	**References**
**As**	Low concentrations in all environmental segments; released through soil erosion and volcanic eruptions	Coal combustion; pesticide formulations; smelting; wood preservative; metal refining; drugs/medicines to treat amoebic dysentery and syphilis; veterinary drugs to treat against parasitic diseases	Dermal; ingestion of contaminated food and water; inhalation of contaminated dust; accidental and occupational exposure; wood preservation; pesticide application and manufacturing; glass manufacturing	Carcinogenic; cardiovascular and neurobehavioral disorders; diabetes	Enzymatic biomethylation of inorganic As to its intermediate monomethylarsonic acid, a potential carcinogen; As(III) has the potential to inactivate approximately 200 enzymes; As(V) can substitute phosphate; inhibit repair mechanism of DNA; cellular respiration inhibition	2,27–29
**Cadmium**	Earth's crust, especially sedimentary rocks and water; small amounts in crustaceans, potatoes, leafy vegetables, mushrooms, etc.	Production of batteries, alloys, pigments, fertilizers, pesticides; welding; mining; combustion of fossil fuels and municipal wastes; recycling of electronic and cadmium-plated waste	Ingestion of contaminated food; inhalation (smoking); occupational exposure	Acute and chronic exposures lead to lung and stomach cancer; ingestion leads to vomiting, pain, nausea, renal injury, consciousness loss, coma; acute inhalation damages lung tissues and causes chest pain, fever, tachycardia; in vitro/in vivo studies have reported genotoxic effects in animals; cadmium exposure leads to chromosomal aberrations; chromosomal damage; multi-organ dysfunction including liver kidney, lungs and heart; kidney toxicity leads to irreversible proteins loss in urine; osteo-toxicity due to bone resorption or inhibiting bone formation; osteoporosis anemia	DNA damage; interruption of protein and nucleic acid synthesis; blocking repair mechanisms; initiation of cellular multiplication; complex formation with metallothionein causing nephrotoxicity	2,30–35
**Cr**	Low concentrations in all environmental segments (Cr (II) to Cr(VI) forms)	Metal processing; dyeing; leather tanning; pigments; chrome plating; wood preservation; metallurgy; welding; boilers and cooking systems as anti-corrosives	Ingestion of Cr contaminated food items and water; inhalation; dermal	Cr(VI) is the main toxic form; dermatitis; kidney damage; asthma, allergies; respiratory tract cancer; inhalation causes ulcers of nose; ingestion causes severe gastrointestinal; cardiovascular, renal, respiratory and neurological disorders; carcinogenic	Cr(VI) causes chromosomal aberrations and DNA strand breaks	2,36,37
**Lead**	Occurs in earth's crust (soil)	Lead batteries, soldered metal products; X-ray shields; glass manufacturing, paints and pigments; ammunition; coal combustion	Ingestion of contaminated water and food; inhalation of leaded dust	Anemia, appetite loss; systemic toxicity has multi-organ effects: kidney, liver and central nervous system reproductive and gastrointestinal systems	Mimics calcium and inhibits body calcium metabolism and cycling; interrupts synthesis and repair of DNA and tumor suppressor proteins	2,38
**Mercury**	Environmental occurrence in water, soil and air	Electrical industries (switches, batteries); paint industry; dentistry; mining	Ingestion, inhalation and dermal	Absorbed by the gastrointestinal tract and crosses blood-brain and placental barriers; accumulates in the kidneys, liver and the nervous tissue; displays neurotoxic, gastrointestinal toxicity and nephrotoxic effects	Bonds covalently with proteins and exhausts antioxidants	2

Abbreviation: DNA, deoxyribonucleic acid.

## Discussion

Rapid population growth has created excessive pressure on terrestrial and aquatic ecosystems, leading to increasing exploitation/extraction of water, food, and water resources. Apart from anthropogenic discharge of heavy metals, natural sources contribute significantly to heavy metal pollution. Their occurrence in soil and release due to soil weathering is an important source of heavy metal pollution. Arsenic is present in various igneous and sedimentary rocks in high concentrations. Approximately 45,000 tons of As is reported to be released by coal sources.^28^,^39^ Many countries, including India, Pakistan, Australia, Canada, Nepal, and Japan are severely impacted by As occurrence.^40^ Arsenic contamination from geogenic sources has been found throughout West Bengal, India.^39^The bioavailability of heavy metals and their effects further depend on the metal, its physico-chemical properties and lipid solubility which imparts a characteristic toxicological property. Features like age, nutritional status, trophic interactions and physiological adaptations of organisms play an important role in their toxicity. With absorption, a metal is distributed in body tissues and tends to persist in the body in organs such as bones, liver and kidneys for a prolonged time. Heavy metals have been reported to affect cellular fractions and organelles.^2^ With increasing awareness regarding the persistence, nature and deleterious effects of heavy metals, there has been growing interest in the development of technologies to remediate this contamination.

### Heavy metals removal through conventional techniques

Conventional techniques like adsorption, electro-dialysis, precipitation and ion exchange used to remove heavy metals have limitations. The process of chemical precipitation involves adding anions for precipitating metals as suspended particles, which are then removed. The process is not specific and cannot remove heavy metals at low concentrations.^41^ Through the ion exchange process, heavy metals can be removed to the level of parts per billion.^42^ However, it's a non-specific, pH-sensitive and expensive method.^43^ The method of reverse osmosis makes use of membranes. These conventional techniques have drawbacks such as slow and inefficient removal, generation of contaminated sludge requiring careful disposal, high cost and energy involved in the processes, and blockage of membranes.^44–47^ There is a need for a cheap and effective technology to remove heavy metals with an eco-friendly approach. There has been increasing interest in the use of biological agents for heavy metal removal as an alternative to these methods.

### Bioremediation

Microorganisms are ubiquitously present in nature and play a crucial role in elemental biogeochemical cycles of metal transformations between soluble and insoluble species. Metal-microbe interactions can have beneficial or harmful consequences. Apart from the nature of the microbes and chemistry of metals involved, these transformations are dependent on other environmental factors like pH, moisture, temperature, presence of other ions, humic colloidal substances and other living organisms and their competitors which play an important part in microbial colonization and biofilm formation.^36^

**Figure 3:**
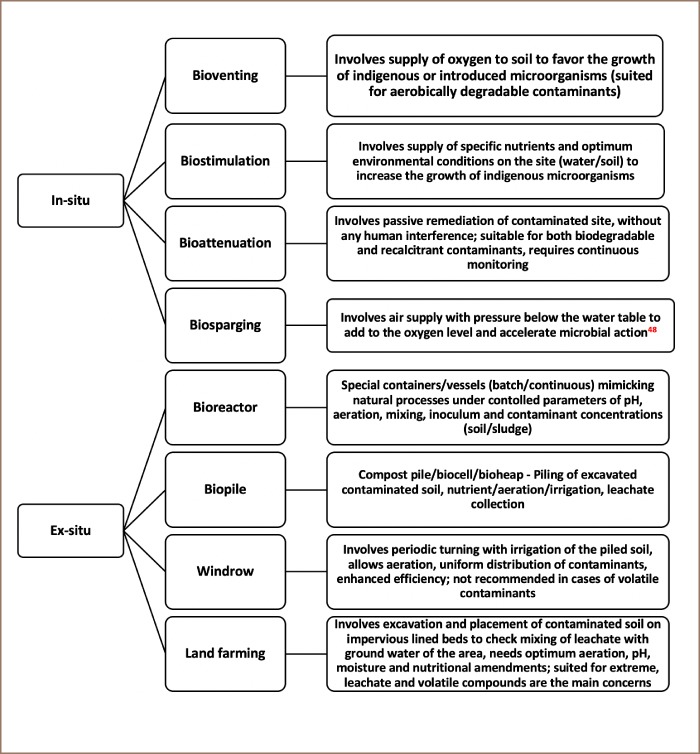
Types of bioremediation techniques for various contaminants^49^

Bioremediation is a technique for removing/converting harmful contaminants like heavy metals into less harmful substances; and/or removing toxic elements from the contaminated environment; or degrading organic substances and ultimate mineralization of organic substances into carbon dioxide, water, nitrogen gas, etc., employing dead or alive biomass. The process of bioremediation can be applied to soil and water media through in- and ex-situ techniques. A brief description of the different techniques of bioremediation for various contaminants is given in Figure 3.

The in-situ process does not involve excavation or removal and does not disturb the soil structure. It can make use of stimulation of indigenous microbial flora (intrinsic) or introduction of microorganisms (engineered) bioremediation.^50^ The type and the nature of contaminants, degree of contamination, soil type or site geochemistry and geographical location are some factors to be considered for this technique.^51^ Contaminants may get adsorbed on soil particles and become unavailable for bioremediation. During bioremediation there are other challenges, like microbial competition and death after inoculation, temperature, and moisture condition of the media.^52–54^ High temperature increases the solubility of contaminants and hence their mobilization.^55^ In-situ treatment generally involves pumping oxygen/nutrients (bioventing/biostimulation) into the soil. The texture also plays an important part during bioventing and biostimulation. In coarse-textured soils, it is easier to pump and disperse oxygen and nutrients compared to fine-textured soils. Fine soils like clay retain moisture in their numerous smaller pores with high surface area and prevent oxygen from dispersing uniformly throughout the contaminated soil. However, this process may not be suitable for all types of soils when natural conditions (like temperature) become limiting. Higher sorption capacity by microbial cells compared to clay particles has been indicated by various reports.^56^ Indigenous species isolated from contaminated sites has been reported to demonstrate exceptional resistance and biosorption efficiency towards heavy metals. Two indigenous strains, AK1 and AK9, belonging to the genus of Pseudomonas, have been isolated from As-contaminated water of the Ganga basin. The strains have been reported to be resistant towards As and other heavy metals, like silver (Ag), cadmium (Cd), cobalt (Co), Cr, Cu, mercury (Hg), Ni, and lead (Pb).^57^ Ex-situ technique involves the transport of contaminated soil and water from the contaminated area to another site for further treatment. It may be classified as a solid-phase technique (for land treatment), slurry-phase and pile techniques (for a mixed medium containing solid and liquid phases in bioreactors).^58^ It uses techniques like bioreactors, biopiles, and land farming. For the slurry-phase technique, contaminated soil is mixed with water along with other additives in a bioreactor. However, the efficiency of the bioreactor depends on biosorbent (live/dead), optimal conditions required for microbial growth and adaptability of biomass to the configuration of the bioreactor.^59^

Mercury removal from synthetic wastewater using a bioreactor has been reported.^60^ The wastewater bioremediation is dependent on various factors like pH.^42^ The pH affects their bioavailability by influencing the solution chemistry through processes like complexation, hydrolysis, redox and precipitation.^61^ Microbial biomass surface area and pretreatment processes (modifying the surface area) tend to influence the bioremediation process.^62^ Microbial biomass may be required to be immobilized in matrices like alginate and silica gel to develop a suitable commercial biosorbent with appropriate strength and porosity.^63^ Encapsulation imparts physicochemical stability and heat resistance. Encapsulated Agrobacterium sp. in alginate with nano-particles of Fe has shown an excellent adsorption capacity for continuously five cycles.^64^ Poor selectivity and difficulties in reusing biomass are some of the limitations of the process. Bioremediation has also been mediated through microbial biofilms having high resistance and tolerance for metal ions. Rhodotorula sp. have a removal efficiency of up to 95.39%.^65^

### Biosorbent materials

Selecting an efficient, highly selective and economical biosorbent is a major concern.^63^ The biosorbent should be easily available or should demonstrate quick growth. The efficiency of the biosorbent depends on the experimental requirements and pretreatment of the bioagent. There have been many reports on wastewater treatment using various biosorbents.^66–72^ Various types of biological agents have been employed for remediation in ex- and in-situ conditions. These include agro-wastes like wheat/rice straw, tea/coffee/yeast waste, cotton waste, etc. Microorganisms (bacteria, fungi, yeast or algae) sourced from their natural habitats can be excellent biosorbents.^73^ These biosorbents can absorb heavy metals at very low concentrations. The functional groups like amide, amine, carbonyl, carboxyl, etc. facilitate the removal of heavy metals. Microorganisms possess characteristic enzymatic profiles required specifically for heavy metal resistance.^74^,^75^ However, steric and conformational factors along with the number and availability of reactive sites affect the biosorption process. Microorganisms like fungi convert heavy metals into less toxic compounds and utilize them for their growth; e.g., Pleurotus sp., Klebsiella oxytoca, etc. display metal binding capacity.^76^,^77^ Cephalosporium aphidicola has been found to be effective in lead-contaminated soil.^78^ Some of the most commonly used biosorbents are shown below in Table 3.

**Table 3:** Different Types of Organisms as Biosorbents (Adapted)^79^

**Biosorbent**	**Examples/species**
Agricultural residues	Rice/wheat straw
Algae	Fucus, Chlamydomonas, Cladophora, Spirogyra sp.
Bacteria	Bacillus sp., Vibrio sp., Geobactor sp., Pseudomonas sp., Desulfovibrio sp.
Fungi	Pleurotus sp., Aspergillus sp., Penicillium sp., Rhizopus sp., Saccharomyces sp.
Industrial wastes	Fermentation/food/beverage wastes
Others	Chitosan- and cellulose-based substances

### Bioremediation mechanisms

Microorganisms adapt to and resist heavy metals in highly contaminated areas. Extra-cellular polymeric substances present on the biomass cell wall can attach to heavy metals by mechanisms like proton exchange or micro-precipitation of metals.^80^ Biomass surfaces have a negative charge because of the presence of carboxyl, amino, phosphoryl, and sulfo groups as potential ion exchange sites and metal sinks. The process of bioremediation takes place through various mechanisms like redox process, adsorption, complexation, ion-exchange, precipitation, and electrostatic attraction.

Microorganisms may initiate metal mobilization/immobilization by redox reactions; and hence, impact bioremediation processes. Heavy metals like Fe, As, Cr, and Hg undergo oxidation and reduction cycles. Bioremediation is facilitated by converting an element from its insoluble and stationary form in sediments into its mobile and soluble phase. Mobilization can also have deleterious impacts when toxic metal ions are redistributed and released from their solid phase from sediments into the solution phase.^59^ This increases their bioavailability and heavy metals can reach microbial metabolic systems. The bacteria reduces Hg(II) to the elemental and more volatile form of Hg(0).^81^ Microbial reduction can also enhance the solubility of ions like Fe(III) and As(V) by reducing them to Fe(II) and As(III), respectively, and can facilitate leaching from soil.^82^,^83^ Studies have reported bacteria from different natural aquifers which can transform As.^84–86^ Pokhrel and Viraraghavan employed Aspergillus niger to remove As(V) and As(III).^87^ Heavy metal biomethylation is an important process in soil and water and may modify toxicity, volatility, and mobility of heavy metals. It also serves as an important means of detoxification as volatile methylated species can be removed from cells.^88^ Dimethylmercury and alkyl arsines, the methylated products of Hg and As, respectively, are volatile and evaporate and are lost from soil. The organic matter fraction of soil serves as the methyl donor. Yet another indirect mechanism of metal mobilization involves the microbial decomposition of organic matter, which accelerates the release of these ions. Schizophyllum commune has been found to release heavy metals along with dissolved organic matter.^89^ Excretion of metabolites like carboxylic acids and amino acids by microbes is an important mechanism of chelating metal ions.

Microbes perform metal immobilization and act as sinks for metals by adopting different mechanisms (ex- or in-situ) like biosorption, bioaccumulation, bioconversion and/or inter/intracellular precipitation (as oxalates of Zn, Cu, Co, Cd, Ni) operating in different ways.^36^,^90^,^91^ By immobilization, an element can be easily removed from its aqueous phase in groundwater or wastewater.^36^,^92^ Bacterial oxidation of As(III) to As(V) makes them immobilized and retained by the sediments.^82^,^83^Methanothermobacter thermautotrophicus has been employed to reduce Cr(VI) to Cr(III) and immobilize it in hydroxide-/oxide-forms. 93 Bacterial reduction and immobilization for Cr(VI) has also been reported for Bacillus cereus and Shewanella sp.^94^,^95^ Cellular structures like the cell wall and plasma membrane act as barriers and check the entry of metal ions into cells.^96^

### Biosorption and bioaccumulation

Biosorption and bioaccumulation are attractive options to substitute conventional methods for heavy metal remediation. Bioaccumulation involves heavy metal uptake by living biomass (metabolism dependent/active uptake) and is characterized by the uptake of contaminants by living biomass/cells. Employing living biomass for remediation may not be a viable option owing to highly toxic metals which can accumulate in cells and interrupt metabolic activities resulting in cell death. However, dead biomass (biosorption) remains unaffected by toxicity, does not require any growth/nutritional medium and is flexible to environmental conditions. Heavy metals are adsorbed on the surface in a passive mode without involving energy expenditure (independent of metabolism) until equilibrium is achieved.^97^ Therefore, biosorption is advantageous, compared to active uptake/bioaccumulation, as it is metabolism independent, however is it largely dependent on the biomass/biosorbent type and contaminants involved.^98^ For these advantages, microbial biomasses of fungi, algae or yeast have been utilized for bioremediation for in-situ processes. Heavy metal bioremediation in the form of metallic nanoparticles with the help of bacteria and the use of genetically modified microorganisms as a part of the bioremediation process have also been reported. 99–101

Intracellular sequestration is the concentration of metal ions within the microbial cells. It involves complexation of heavy metal ions due to surface interactions and their subsequent transport into the cell.^96^ Extra-cellular sequestration comprises a concentration of metal ions in the periplasm or their complexation as insoluble precipitates. Cadmium precipitation has been reported in Pseudomonas aeruginosa and Klebsiella planticola.^102^,^103^

### Bacterial bioremediation

Bacteria are ubiquitously present in the environment. Bacteria are found in different shapes, including rods (Bacillus), cocci (Streptococcus), filamentous (Actinomyces) and spiral (Vibrio cholera). Biosorption by bacteria is an inexpensive and efficient technique to remove pollutants, including non-biodegradable elements, like heavy metals, from wastewater. Bacterial biomass can be living or non-living cells. Bacterial species have adapted and developed mechanisms for metals ions resistance and remediation for their survival.^104^ Heavy metal ions bioremediation by bacterial agents has been widely researched.^105–109^ Bacterial biomass accomplishes the rapid removal of metals such as Cu, Zn, Pb, Cd, and Cr.^110^ biosorption efficiency depends on heavy metal ions and bacterial species (owing to their different cellular structures in terms of peptidoglycans like N-acetylmuramic acid and poly-N-acetylglucosamine). 42 The bacterial cell wall is the primary physical contact linking metal ions and the bacterial biomass. The overall negative charge due to anionic functional groups (like amine, hydroxyl, carboxyl, sulphate, phosphate) present in Gram-positive bacteria (in peptidoglycan, teichoic acids, and teichuronic acids) and in Gram-negative bacteria (in peptidoglycan, lipopolysaccharides, and phospholipids) imparts metal-binding capacity on or within the cell wall.^111^ The heavy metal removal by dead biomass cells is extracellular. Functional groups, including carboxyl, phosphonate, amine and hydroxyl groups on the cell wall are responsible for these interactions.^112^,^113^

The carboxyl groups can bind Cd on the surface by complexation.^114^ The amino groups have displayed efficient removal of Cr by chelation and electrostatic interactions.^115^ Bacterial species need to be exposed to the contaminants for enzymatic induction before using them for bioremediation. There is a minimum requirement of contaminant concentration to initiate enzymatic expression necessary for the process.^116^ Species like Pseudomonas, Desulfovibrio, Bacillus, and Geobacter have been used for bioremediation *(Table 4)*.

**Table 4:** Biosorption by Bacterial Species

**Bacterial species**	**As**	**Cd**	**Cr**	**Co**	**Cu**	**Hg**	**Ni**	**Pb**	**Zn**	**Reference**
**Arthrobacter sp.**					x					117
**Bacillus sp.**					x			x		118
**Bacillus sp.,**	x									119
**Aneurinibacillus sp.**										
**Bacillus laterosporus**		x	x							120
**Corynebacterium glutamicum**								x		121
**Desulfovibrio desulfuricans**			x		x		x			122
**Bacillus sp., Pseudomonas sp., Micrococcus sp.**		x		x				x		123
**Bacillus licheniformis**			x				x			124
**Geobacter metallireducens**	x									125
**Geobacter sulfurreducens**			x							126
**B. licheniformis**			x		x					127
**Klebsiella planticola**		x								103
**Micrococcus luteus**					x			x		128
**Pseudomonas aeruginosa**		x								102,129
**Pseudomonas fluorescens**	x	x	x	x	x	x	x	x		130
**Pseudomonas putida**		x			x			x	x	131,132
**P. aeruginosa**			x							115
**P. aeruginosa**	x									133
**Rhizopus arrhizus**			x							134
**Rhodopseudomonas palustris**				x						135
**Streptococcus equisimilis**			x							136
**Staphylococcus xylosus**	x									137
**Vibrio harveyi**								x		138

Abbreviations: As, arsenic; Cd, cadmium; Cr, chromium; Co, cobalt; Cu, copper; Fe, iron; Hg, mercury; Mn, manganese; Ni, nickel; Pb, lead; Zn, zinc.

### Algal bioremediation (phycoremediation)

Different species of algae are present in large amounts in marine ecosystems. Algae are autotrophic organisms, have low nutritional requirements and generate vast biomass.^79^ Among the three algal groups; i.e., Phaeophyta, Rhodophyta and Chlorophyta (i.e. brown, green and red, respectively), brown algae have been reported to possess better biosorption capacity (phycoremediation). Metal ion biosorption varies with the kind and structure of the algal biomass, charge and chemical constitution of the heavy metal ion.^139^,^140^ Different algae, in live or dead forms, have been used, as single or in combination, in batch or column, for in-situ remediation. The presence of amine, hydroxyl, carboxyl, sulphate, and phosphate are potential metal sites in algal proteins, which operate by complex formation methods during heavy metal remediation.^79^,^141^ Calcium, magnesium, and sodium ions present in the cell wall get replaced by heavy metal ions via ion exchange. Table 5 depicts various types of algae as biosorbents.

**Table 5—:** Biosorption by Algal Species

**Algal species**	**As**	**Cd**	**Cr**	**Co**	**Cu**	**Fe**	**Hg**	**Mn**	**Ni**	**Pb**	**Zn**	**Reference**

**Asparagopsis sp., Codium sp., Spirogyra sp., Chondrus sp.,Fucus sp., Ascophyllum sp.**		x			x				x	x	x	141
**Ceramium virgatum**		x										142
**Chlamydomonas reinhardtii**		x					x			x		143
**Chlorella vulgaris**		x							x	x	x	144
**C. vulgaris**		x			x					x		145
**C. vulgaris, Scenedesmus armatus**						x		x			x	146
**Chlorella kessleri**					x						x	147
**Corallina mediterranea, Galaxaura oblongata,**		x	x	x						x		148
**Jania rubens, Pterocladia capillaceaCladophora fascicularis**										x		149
**Caulerpa fastigiata**		x										150
**Caulerpa lentillifera**		x			x					x		151
**Spirogyra hyalina**	x	x		x			x		x	x		152
**Fucus vesiculosus**		x			x					x		153
**F. vesiculosus**					x							154
**Isochrysis galbana**			x									155
**Pithophora oedogonia**					x					x		156
**Sargassum polycystum**			x									157
**Sargassum sp., Padina sp., Ulva sp., Gracillaria sp.**		x			x				x	x	x	158
**Scenedesmus quadricauda**				x					x		x	159
**Schizosaccharomyces pombe**					x							160
**S. hyalina**	x	x		x			x			x		161
**Spirogyra sp.**		x			x			x			x	162
**Spirulina platensis**		x										163
**Ulva fascia**					x				x			164
**Ulva lactuca, Jania rubens, Sargassum asperifolium**										x		165

Abbreviations: As, arsenic; Cd, cadmium; Cr, chromium; Co, cobalt; Cu, copper; Fe, iron; Hg, mercury; Mn, manganese; Ni, nickel; Pb, lead; Zn, zinc.

### Fungal bioremediation (mycoremediation)

Fungi are known for their pervasive presence in the natural environment and are exploited extensively in industrial applications.^166^ Fungi are adapted (in terms of their morphology, ecology and metabolism) according to environmental conditions and are responsible for processes like decomposition and nutrient cycling under natural conditions.^167^ They have been reported to withstand and survive under stress conditions of moisture, nutrients, pH, etc. Mycoremediation involves use of fungus (live or dead) for the removal of contaminants from different environmental segments.^168^,^169^ Mycoremediation is a cost-effective process and does not leave harmful waste products. Hence, it poses a complete solution because of the full mineralization of the pollutants in nature.^170^ The success of mycoremediation depends on the identification and usage of a suitable fungal species for the target heavy metal or other contaminants. Fungi have the ability to accumulate heavy metals in their fruit bodies in an efficient manner, making them unavailable or decreasing their concentration in the media.^171^ The future availability of heavy metals and other contaminants in the media depend upon the life of the fungi, chemical behavior of the elements and presence or absence of the fungi after sequestration. Saccharomyces cerevisiae has been reported to sequester up to 65–79% of Pb and Cd from polluted soil.^172^ The process of biosorption involves fungal cell walls (having chitin, proteins, glucans, lipids, pigments, polysaccharides) and functional groups like hydroxyl, carboxyl, amino, sulphate, or phosphate and is mediated through interactions like adsorption, ion-exchange and complexation.^173–175^ Aspergillus sp. have been reported to remove Cr from tannery wastewater; it removed 65% of the Cr from the wastewater as compared to 85% from the synthetic medium.^176^

The phylum basidiomycetes includes wood-decaying species (white- and brown-rot fungi), mushrooms and other fungi.^177^ Mushrooms have played an important role in the human diet throughout history due to their nutritional and medicinal properties. Besides their use as food, they are used for mycoremediation due to their potential for heavy metal uptake. Metal uptake in mushrooms is affected by contact time, age of mycelia and fructification.^178^ Some edible wild varieties of mushrooms can accumulate heavy metals over their background concentrations.^179^ Heavy metals scatter disproportionately in the mushroom fruiting body.^180^,^181^ Different species of white-rot fungi, including Pleurotus ostreatus and Termitomyces clypeatus have been reported to degrade persistent pollutants *(Table 6)*.^181^,^182^

**Table 6:** Bioremediation by Fungal Species

**Fungal species**	**As**	**Cd**	**Cr**	**Co**	**Cu**	**Fe**	**Hg**	**Mn**	**Ni**	**Pb**	**Zn**	**Reference**

**Agaricus bisporus, Pleurotus ostreatus**			x		x					x		183
**A. bisporus**		x	x	x						x		184
**Alternaria alternata, Penicillium aurantiogriseum**		x					x					185
**Aspergillus sp.**									x			186
**Aspergillus flavus, Aspergillus fumigatus, Paecilomyces sp., Cladosporium sp., Mucor sp.**	x											187
**A. flavus, A. gracilis, A. penicillioides, A. restrictus, Sterigmatomyces halophilus**		x			x	x		x		x	x	188
**Aspergillus sp., Rhizopus sp.**		x	x									189
**Aspergillus sp., Penicillium sp.**		x										190
**Schizophyllum commune**			x		x				x		x	191
**P. ostreatus**			x		x	x					x	192
**Pleurotus ferulae**										x		193
**P. ostreatus**										x		194
**Pleurotus floridaTrichoderma viride**										x		195
**Rhizopus nigricans**										x		196
**Rhizopus arrhizus**			x									197
**Penicillium coffeae**	x											198
**A. flavus, A. fumigatus, Helminthosporium sp., Cladosporium sp., Mucor sp.**							x					199
**Penicillium janthinellum**	x											200
**Termitomyces clypeatus**			x									201
**Talaromyces helicus**					x							202
**Trichoderma viride**			x									203

Abbreviations: As, arsenic; Cd, cadmium; Cr, chromium; Co, cobalt; Cu, copper; Fe, iron; Hg, mercury; Mn, manganese; Ni, nickel; Pb, lead; Zn, zinc.

### Recommendations

The unregulated discharge of industrial effluents in agricultural fields or water bodies increases their chances of entering the food chain through crops and aquatic animals and subsequent bioaccumulation. Various in-situ and ex-situ methods of bioremediation suitable to different environmental conditions have been investigated and recommended.^204–207^ The design, development, and application of these techniques require careful selection of biological agents. Extensive research is being carried out using specific strains of microorganisms for bioremediation. Microorganisms carry out redox reactions; and hence, impact the bioremediation processes by metal mobilization/immobilization. Manganese(II)-oxidizing Bacillus sp. strain indirectly oxidizes Cr(III) into the mobile and bioavailable form of Cr(VI) by producing oxidized Mn.^208^ The process of heavy metal bioremediation is more efficient using different microbial strains concurrently instead of only a single species.^63^ Advances in genetic engineering and optimization techniques suggest that the future of these technologies is promising.^209^,^210^ Genetically modified microorganisms may have a better bioremediation potential for various contaminants. In addition, the potential of agricultural and industrial waste biomass as bioremediators on a lab/commercial scale is currently being tested; e.g., sugarcane bagasse, coconut shell waste, rice husk, and beer waste yeast.^211–214^ The biosorption capacity of various biosorbents is improved after various physical and chemical modifications and further research is needed in order to use these biosorbents on a commercial scale across industries. The bioremediation approach requires a holistic and inclusive method for systematic, feasible and sustainable strategies which can be easily customized for each scenario. Moreover, there is an urgent need for coordination at all levels, including research organizations, the general public, governmental institutions, and industries.^215^

## Conclusions

Heavy metal pollution occurs from various anthropogenic and natural sources. Heavy metals, due to their non-biodegradable and hazardous nature, should be removed from the environment. Industrial wastewater discharged into environmental segments, such as soil and rivers, requires immediate intervention by governmental agencies, regular monitoring and remediation using appropriate methods. Conventional methods of treatment have limitations and should be replaced by efficient, cost-effective and eco-friendly techniques such as bioremediation employing biological agents. Adopting an appropriate biosorbent in terms of efficiency and economy is a major concern. Microorganisms carry out redox reactions; and hence, impact the bioremediation processes by metal mobilization/immobilization. Metal-microbe interaction influences microbial processes, such as their growth, colonization and microbial biofilm formation for remediation. Further research is needed translating these lab scale options into industrial applications (e.g. microbial films) considering factors such as appropriate and adapted microorganisms/appropriate biomass waste and/or mixtures of different kinds of microbial biomass with conventional technologies and suitable conditions in ex- or in-situ environments.
